# Machine Learning-Based Toothbrushing Region Recognition Using Smart Toothbrush Holder and Wearable Sensors

**DOI:** 10.3390/bios15120798

**Published:** 2025-12-05

**Authors:** Hsuan-Chih Wang, Ju-Hsuan Li, Yen-Chen Lin, Che-Yu Lin, Chien-Pin Liu, Tzu-Han Lin, Chia-Tai Chan, Chia-Yeh Hsieh

**Affiliations:** 1 Department of Biomedical Engineering, National Yang Ming Chiao Tung University, Taipei City 112, Taiwan; wxuanzhi.be11@nycu.edu.tw (H.-C.W.); yeach52639.y@nycu.edu.tw (J.-H.L.); casper871116.be10@nycu.edu.tw (Y.-C.L.); jlcy025.be13@nycu.edu.tw (C.-Y.L.); henry062439.be09@nycu.edu.tw (C.-P.L.); tzulyn.be12@nycu.edu.tw (T.-H.L.); 2 Bachelor’s Program in Medical Informatics and Innovative Applications, Fu Jen Catholic University, New Taipei City 242, Taiwan

**Keywords:** oral hygiene, toothbrushing monitoring, toothbrushing region recognition, wearable sensor, machine learning

## Abstract

Oral health is a critical factor in maintaining overall health, and its association with systemic diseases, including cardiovascular disease and diabetes mellitus, has been extensively investigated. Effective plaque removal through proper toothbrushing techniques is fundamental for preventing dental caries and periodontal diseases. Despite standardized guidelines, many individuals fail to adhere to correct brushing techniques, thereby increasing the risk of oral diseases. To address this issue, this study proposes a fine-grained toothbrushing region recognition approach incorporating six machine learning classifiers and two inertial measurement units (IMUs), which are embedded in the toothbrush holder and mounted on the right wrist of the participant, respectively. By analyzing the continuous motion signals, the proposed hierarchical approach is capable of identifying brushing and transition activities and subsequently recognizing specific toothbrushing regions based on the predicted brushing activities. To further improve recognition reliability, post-processing strategies such as contextual smoothing and majority voting are applied. Experimental results demonstrate that random forest achieves the highest recognition accuracy of 96.13%, sensitivity of 96.10%, precision of 95.51%, and F1-score of 95.60%. The results indicate that the proposed approach is both effective and feasible for providing fine-grained toothbrushing region recognition in toothbrushing monitoring.

## 1. Introduction

Oral health plays a crucial role for humans in performing essential functions, including chewing, swallowing, speaking, and maintaining overall health. Based on previous studies, the relationship between oral health and systemic diseases, such as cardiovascular disease, diabetes mellitus, respiratory disease, and diverse pregnancy outcomes, has been explored [1,2]. Poor oral health may lead to oral diseases such as gingivitis, tooth decay, and periodontal disease. According to estimation of the World Health Organization (WHO), approximately 3.5 billion people worldwide are affected by oral diseases [3]. Among these, gingivitis is the most common and mildest form of periodontal disease, affecting 50% to 90% of adults worldwide [4]. Research has further indicated that periodontal pathogens may accumulate within blood vessels, resulting in atherosclerosis [5]. Additionally, the association between periodontal disease and incident stroke has been confirmed [6]. Given its widespread impact and potential systemic consequences, oral health should not be neglected.

Consistent and regular dental care, particularly toothbrushing, is essential for inhibiting bacterial colonization and plaque formation, thereby maintaining oral hygiene and significantly reducing the risk of oral diseases. One of the most recommended and effective brushing techniques is the modified Bass brushing technique [7,8,9,10]. The Bass Brushing Technique involves four parts. First, brush all surfaces, including the outer, inner, and occlusal surfaces, covering one to three teeth at a time. Second, position the bristles at a 45-degree angle toward the gingival margin when cleaning the outer and inner surfaces. Third, move the toothbrush back and forth parallel to the occlusal surfaces when brushing chewing surfaces. Fourth, repeat the brushing motion for each tooth for approximately 15 to 20 strokes before proceeding to the next tooth. In total, the recommended brushing duration exceeds 120 s, which is generally regarded as the optimal time for effective oral hygiene. Despite the existence of this standardized protocol, few individuals adhere to its recommended procedures. Ganss et al. [11] reported that none of the 103 uninstructed participants employed the modified Bass brushing technique, and only 22.3% of the participants brushed for longer than 120 s. These findings highlight a substantial discrepancy between recommended oral hygiene guidelines and actual daily practices, indicating that improper brushing behaviors remain widespread among the general population. Such deviations from proper technique, including incomplete coverage of all tooth surfaces or uneven distribution of brushing time, are major contributors to dental disease, as they result in inadequate plaque removal [12]. Poor oral hygiene leads to bacterial accumulation on the tooth surface and along the gingival margin, thereby increasing the risk of periodontal disease [13]. These bacteria release toxins that trigger an immune response, causing gingival inflammation, swelling, and tissue damage. If left untreated, the condition can progress to gingival recession and eventual tooth loss. Given the high prevalence of oral diseases and the widespread use of suboptimal brushing techniques [14,15], there is an urgent need to develop a brushing monitoring system that promotes proper brushing behavior and improves oral health outcomes. To address these behavioral limitations and promote effective oral hygiene, brushing monitoring systems have been developed to provide personalized feedback based on users’ brushing behaviors. Such systems aim to help users establish proper brushing habits for efficient plaque removal by considering key factors such as daily brushing frequency, number of strokes per tooth, surface coverage, and brushing duration. Consequently, accurate recognition of brushing regions is both necessary and fundamental.

With the advancement of microelectromechanical systems (MEMS) technology, inertial sensors have been increasingly utilized as signal acquisition devices for brushing region recognition. Chen et al. [16] utilized a smart toothbrush equipped with a built-in inertial sensor to capture the brushing motion signals for recognizing brushing areas and postures. The results demonstrated an average recognition accuracy of 99.08% across 15 regions using a recurrent probabilistic neural network (RPNN) model. Additionally, Haicui and Lei [17] attached an inertial sensor to the toothbrush handle and employed a random forester classifier (RFC) model to detect 16 brushing surfaces and estimate brushing force, achieving an average accuracy of 74.04% for online brushing region detection. Collectively, these studies, along with several previous studies [18,19,20,21,22], have demonstrated the feasibility and effectiveness of using inertial measurement units (IMUs) for brushing region recognition.

However, several limitations and unaddressed issues remain in the existing literature. The overall brushing activity can be decomposed into two types of segments, including brushing segments and transition segments. During brushing segments, the bristles are in contact with a specific dental region and perform the actual cleaning motion. In contrast, transition segments refer to the intervals between brushing segments when the brush head moves from one region to another without making effective contact with the tooth surfaces. As these transition segments do not contribute to dental cleaning, brushing strokes and duration may be overestimated if the transition segments are not properly identified and excluded during the evaluation of brushing adequacy. Hence, the accurate detection of transition segments is essential for the development of reliable brushing monitoring systems. Furthermore, according to the Bass brushing technique, approximately 10 to 20 brushing strokes are recommended for each group of two to three teeth. Consequently, an effective and reliable brushing monitoring system must be capable of classifying fine-grained brushing regions to accurately assess whether the appropriate number of strokes has been applied to each surface. In summary, a fundamental requirement for developing an intelligent brushing monitoring system is the ability to extract brushing segments from IMU signals and to recognize fine-grained brushing regions for the comprehensive assessment of brushing completeness and stroke adequacy.

To enable more precise evaluation of brushing completeness in future monitoring systems, this pilot study focuses on brushing region recognition. A hierarchical approach is proposed for identifying fine-grained brushing regions using IMU-based motion signals. The proposed hierarchical algorithm consists of two sequential models, where the first model identifies brushing and transition segments, and the second model further classifies these brushing segments into specific dental regions.

## 2. Materials and Methods

**Figure 1:**
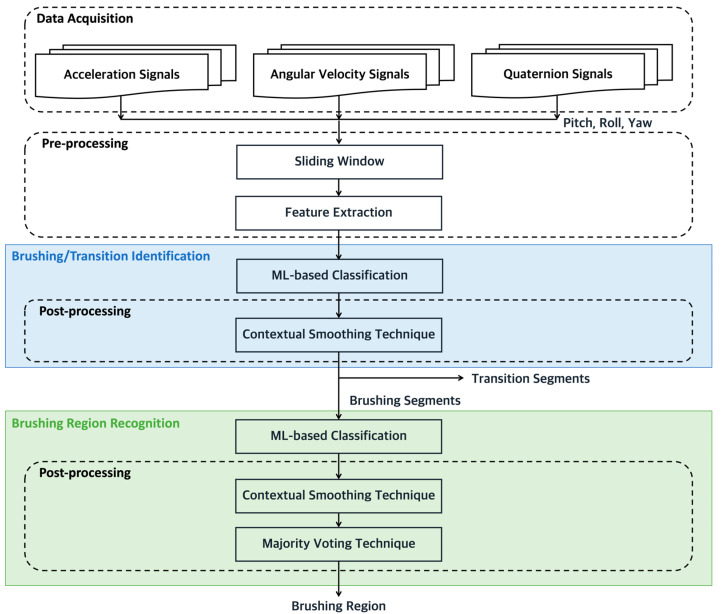
The function diagram of the brushing region recognition.

This study proposes a hierarchical approach to recognize fine-grained brushing regions utilizing IMU-based motion data. The functional diagram of hierarchical algorithm is shown in Figure 1. There are four stages in the proposed method, including data acquisition, signal pre-processing, brushing/transition identification, and brushing region recognition. Firstly, the inertial sensors are employed to capture the movement signals generated during toothbrushing. Secondly, in order to characterize the raw IMU data, the sliding window technique and feature extraction are employed. Finally, machine learning-based (ML-based) classifiers are applied to identify brushing segments and subsequently recognize fine-grained brushing regions. The following sections provide a detailed explanation of each stage.

### 2.1. Data Acquisition

**Figure 2:**
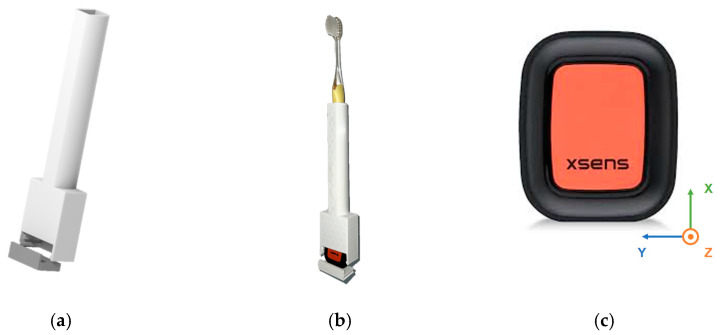
The toothbrush holder and inertial sensors used in the experiment: (**a**) 3D design of the toothbrush holder; (**b**) prototype of the toothbrush holder with embedded conventional toothbrush and sensor; (**c**) The coordinates system of Movella Xsens DOT sensor.

Smart toothbrushes that integrate multiple hardware components are impractical and costly due to the need for regular replacement. Hence, a cost-effective 3D-printed toothbrush holder is developed using DesignSpark Mechanical software 6.0, which was published by RS Components and Electrocomponents plc, Corby, UK, for 3D modeling and fabricated with a Bambu Lab X1 Carbon 3D printer, which was manufactured by Bambu Lab, Shenzhen, China, using polylactic acid (PLA) filament. The toothbrush holder is designed to accommodate a manual toothbrush with a handle cross section of 14 mm × 12 mm and features a dedicated compartment mounting an inertial sensor with a removable lid, as depicted in Figure 2a,b.

**Figure 3:**
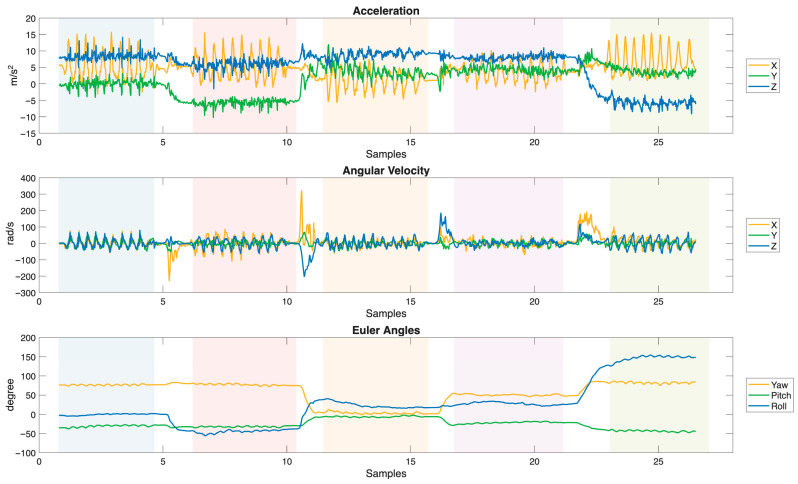
The motion signals of tri–axial acceleration, tri–axial angular velocity, and Euler angles.

The Movella Xsens DOT sensors, which were manufactured by Movella, Enschede, The Netherlands, are utilized to acquire the motion signals corresponding to distinct toothbrushing behaviors, as shown in Figure 2c. Each sensor is equipped with a tri-axial accelerometer (range: ±16 g), gyroscope (range: ±2000 deg/s), and magnetometer (range: ±8 Gauss), operating at a sampling rate of 60 Hz. Tri-axial acceleration from the accelerometer, tri-axial angular velocity from the gyroscope, and Euler angles converted from quaternions are further processed in the subsequent stage, and the motion signals are illustrated in Figure 3. For data acquisition, a smartphone application (Movella DOT app) is used to collect and store sensor data. In addition, a synchronized camera is employed to simultaneously record video data for data labeling. Two sensors are worn on the right wrist of the participant and embedded in the toothbrush holder, respectively. In consideration of future practical deployment, we compare the recognition performance of brushing regions under different sensor placements and configurations.

### 2.2. Experimental Protocol

**Figure 4:**
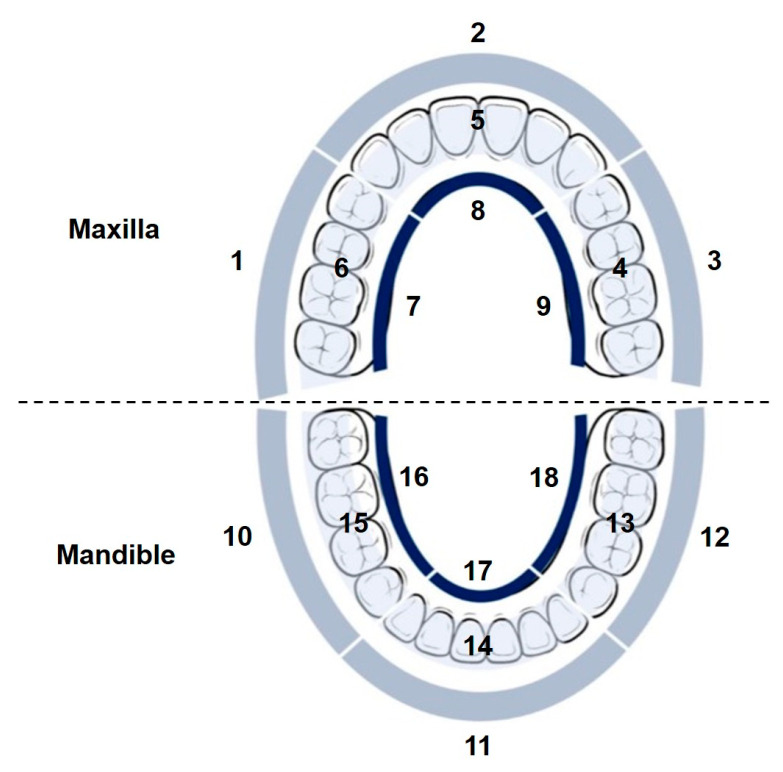
Subdivision of 18 brushing region.

According to the Bass Brushing Technique, each group of two to three teeth should receive approximately 10 to 20 brushing strokes across the occlusal, buccal and lingual surfaces to ensure effective plaque removal and maintain oral hygiene. However, due to the limited space within the oral cavity, it is challenging to distinguish small-scale actions or specific regions based on micro-motions and the subtle variations in IMU signal patterns. As a pilot study toward detailed recognition of brushing regions, the proposed method adopts an 18-region subdivision of the dentition, which provides finer resolution than the coarse segmentation schemes commonly used in previous research, as depicted in Table 1 and Figure 4. Before the experiment, the participant was instructed on proper brushing techniques and the designated sequence of brushing regions, including the starting point and the prescribed order of progression. The specified sequence involves the maxillary regions in the order 1→2→3→4→5→6→7→8→9, followed by the mandible regions in the order 10→11→12→13→14→15→16→17→18, with each region receiving 20 brushing strokes. A complete toothbrushing session is defined as a sequence that covers all brushing regions in a specified order without repetition. The participant is instructed to perform no more than three sessions per day over a two-week period, resulting in a total of 30 recorded sessions from a subject. This study was approved by Institutional Review Board of National Yang Ming Chiao Tung University (protocol code: NYCU114010AE).

### 2.3. Signal Pre-Processing

In the signal pre-processing stage, the sliding window technique and feature extraction are employed to characterize the IMU signals. In order to comprehensively represent the motion magnitude, the following 14 signal dimensions are extracted, as detailed in Equations (1)–(4): (1) raw triaxial acceleration and angular velocity; (2) Euclidean norms of triaxial acceleration and angular velocity; (3) Euclidean norms of acceleration and angular velocity projected onto the horizontal, coronal, and sagittal planes.

Equation (1) defines the Euclidean norms of triaxial acceleration or angular velocity:

(1) $$o_{norm}=\sqrt{{o_{x}}^{2}+{o_{y}}^{2}+{o_{z}}^{2}},$$

where o represents either acceleration (a) or angular velocity (ω). If o=a, then $a_{x}$, $a_{y},$ and $a_{z}$ denote the acceleration components along the x-, y-, and z-axes, respectively. Conversely, if o=ω, then $\omega_{x}$, $\omega_{y}$, $\omega_{z}$ denote the angular velocity components along the x-, y-, and z-axes, respectively.

Equations (2)–(4) define the Euclidean norms of acceleration or angular velocity projected onto the horizontal, coronal, and sagittal planes, respectively:

(2) $$o_{hori}=\sqrt{{o_{y}}^{2}+{o_{z}}^{2}},$$

(3) $$o_{coro}=\sqrt{{o_{x}}^{2}+{o_{z}}^{2}},$$

(4) $$o_{sagi}=\sqrt{{o_{x}}^{2}+{o_{y}}^{2}},$$

where o represents either acceleration (a) or angular velocity (ω).

Subsequently, a sliding window with a size of 20 data points and 50% overlap is applied to segment the continuous motion signals. From each segment, eight common time-domain statistical features, including mean, maximum, minimum, variance, standard deviation, kurtosis, skewness, and range are calculated to characterize the motion signals. A total of 112 features (8 statistical features × 14 signal dimensions) are extracted from segmented signals for each sensor, as shown in Table 2.

Moreover, previous studies [13] have demonstrated that Euler angles offer favorable interpretability and competitive performance in brushing region recognition. To further investigate the impact of Euler angle features on region recognition performance, feature vectors including Euler angles are constructed, as detailed in Table 3, with a total of 136 features (8 statistical features × 17 signal dimensions) are extracted.

Equations (5)–(7) define the Euler angles: pitch, roll, and yaw, which describe orientation in three-dimensional space:

(5) $$Pitch\left(\theta \right)=arcsin\left(2\left(q_{w}q_{y}-q_{z}q_{x}\right)\right),$$

(6) $$Roll\left(\varphi \right)=\arctan\left(\frac{2\left(q_{w}q_{x}+q_{y}q_{z}\right)}{1-2\left({q_{x}}^{2}+{q_{y}}^{2}\right)}\right),$$

(7) $$Yaw\left(\psi \right)=\arctan\left(\frac{2\left(q_{w}q_{z}+q_{x}q_{y}\right)}{1-2\left({q_{y}}^{2}+{q_{z}}^{2}\right)}\right),$$

where $q_{w}$, $q_{x}$, $q_{y}$, and $q_{z}$ are the scalar and vector components of the unit quaternion representing orientation.

### 2.4. Brushing/Transition Identification

In this stage, six machine learning models are utilized to identify the brushing segments and transition segments. The adopted models include support vector machine (SVM), Naïve Bayes (NB), *k*-nearest neighbors (*k*-NN), decision tree (DT), random forest (RF), and adaptive boosting (AdaBoost). To further enhance the performance of brushing segment identification, a contextual smoothing post-processing is implemented. The following sections provide a detailed description of the machine learning algorithms and post-processing method.

#### 2.4.1. Machine Learning-Based Classification for Brushing Segment Identification

- Support Vector Machine (SVM) is a supervised learning algorithm that finds an optimal hyperplane in a high-dimensional feature space to maximize the margin between classes, classifying new data based on their position relative to the separating hyperplane. A linear kernel is employed in this study.
- Naïve Bayes (NB) is a probabilistic supervised learning algorithm based on Bayes’ theorem, assuming conditional independence features. It estimates the class-conditional probabilities from training data and predicts the class with the highest posterior probability.
- k-Nearest Neighbors (*k*-NN) is a supervised learning method that predicts outcomes based on the distance to nearby data points, assigning the labels by majority voting for classification tasks or averaging the values of the k nearest neighbors. In this stage, the parameter *k* = 5 is utilized for classification.
- Decision Tree (DT) is a supervised learning algorithm that recursively partitions data through a hierarchical tree structure, selecting features that maximize class separation. Each internal node represents a test condition on a specific feature, while each leaf node indicates the final predicted class.
- Random Forest (RF) is an ensemble learning method that constructs multiple decision trees on bootstrapped data and random feature subsets to enhance classification performance. The final predictions are obtained by aggregating the outputs of all trees through majority voting for classification tasks or averaging for regression tasks. In this stage, RF model is implemented with 310 trees.
- Adaptive Boosting (AdaBoost) is an ensemble learning algorithm that builds a strong classifier by iteratively combining multiple weak learners. In each iteration, it increases the weights of misclassified samples, thereby encouraging the subsequent weak learner to focus more on difficult instances. The final output is obtained through a weighted majority vote of all weak learners. The maximum number of splits for per tree is set to 250 and the number of learning iterations is set to 150.

#### 2.4.2. Post-Processing for Brushing Segment Identification

Due to the sliding window technique applied to the time-series data, occasional misclassifications may occur at the boundaries between transition and toothbrushing activities. Given the temporal consistency of toothbrushing activities, the model’s predictions are expected to exhibit temporal coherence. To reinforce this property, a contextual smoothing strategy is applied during post-processing.

**Figure 5:**
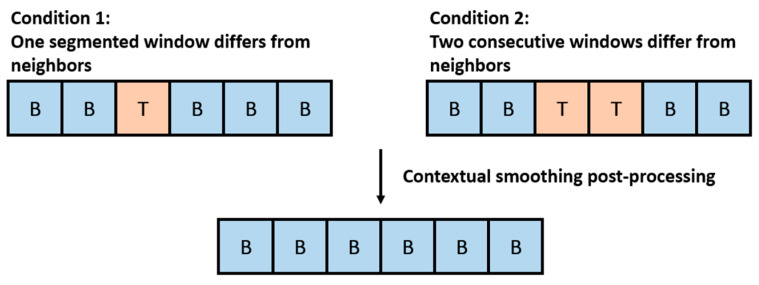
Examples of contextual smoothing post-processing applied to inconsistent predictions, where B represents brushing segments and T presents transition segments.

This technique examines the predicted labels of consecutive segmented windows and corrects isolated inconsistencies based on the surrounding context. Specifically, if one or two consecutive windows are predicted as a different class from their neighboring windows, and the surrounding windows are classified as the same class, the inconsistent windows are adjusted to match the surrounding class. Figure 5 illustrates two different conditions of such inconsistencies, both of which are corrected to yield a consistent result after post-processing.

### 2.5. Brushing Region Recognition

This stage aims to recognize the specific brushing region within each detected brushing segment. The overall structure is similar to the previous Brushing/Transition Identification pipeline, adopting machine learning-based classification followed by a comparable post-processing procedure. A contextual smoothing strategy and a majority voting technique are incorporated in the post-processing step to further enhance recognition accuracy. The machine learning algorithms and post-processing procedure are described below.

#### 2.5.1. Machine Learning-Based Classification for Brushing Region Recognition

The same machine learning models previously used for brushing segment identification are also applied in this stage, including DT, RF with 310 trees, AdaBoost with a maximum of 250 splits per tree and 200 learning iterations, *k*-NN with *k* = 9, NB, and SVM with a linear kernel. The primary difference lies in the number of output classes, as the models are designed to perform multi-class classification to predict one of 18 predefined brushing regions, rather than merely distinguishing between brushing and transition segments.

#### 2.5.2. Post-Processing for Brushing Region Recognition

Misclassifications may occur due to incomplete observation of the target region within individual windows, local variations in brushing motion, or signal noise. Because each window is evaluated independently, isolated incorrect predictions may arise even within a single brushing segment, potentially leading to inconsistencies in the assigned region labels. To enhance consistency, the contextual smoothing strategy is first employed, followed by a majority voting technique during the post-processing stage, differing from the approach described in Section 2.4.2.

**Figure 6:**
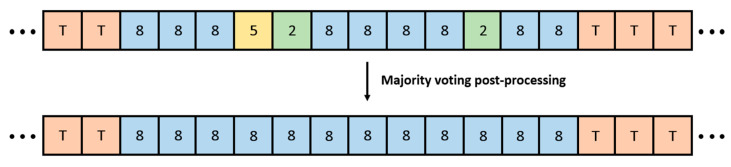
Examples of majority voting post-processing for inconsistent predictions, where T denotes a transition segment and the numbers indicate brushing regions, as shown in Figure 4.

Each brushing segment, defined as the interval between two consecutive transition segments, is assumed to correspond to a single brushing region, and all subsegments within the same segment are expected to share the same region label. Specifically, the final region label for each brushing segment is determined by applying majority voting over all window-level predictions within that segment. As illustrated in Figure 6, the majority voting technique is applied to each segment defined between two consecutive transitions identified during the Brushing/Transition Identification stage. Within each of these segments, the window-level predictions of brushing regions are aggregated, and the most frequently predicted region is selected as the final region label for the entire segment.

### 2.6. Performance Evaluation

In this study, a leave-one-session-out cross validation approach is utilized to validate the performance of the proposed hierarchical brushing region recognition method. Four metrics derived from the confusion matrix are used to evaluate the performance, including accuracy, sensitivity, precision, and F1-score, as defined in Equations (8)–(11):

(8) $$Accuracy=\frac{TP+TN}{TP+FP+TN+FN},$$

(9) $$Sensitivity=\frac{TP}{TP+FN},$$

(10) $$Precision=\frac{TP}{TP+FP},$$

(11) $$F1\text{-}score=\frac{2\times Sensitivity\times Precision}{Sensitivity+Precision},$$

where *TP* denotes the number of segmented windows correctly classified as brushing regions (true positives), *TN* denotes those correctly classified as non-specific brushing regions (true negatives), *FP* denotes the number of windows incorrectly classified as brushing regions (false positives), and *FN* denotes those incorrectly classified as non-specific brushing regions (false negative). Accuracy represents the overall proportion of correctly classified windows among all predictions, providing a general measure of classification performance. Sensitivity indicates the ability of the model to correctly identify true brushing regions, while precision reflects the ability to accurately predict these regions. *F*1-*score* is the harmonic mean of sensitivity and precision. Generally, higher values of these four metrics correspond to superior model performance.

## 3. Results and Discussion

### 3.1. Experimental Signals

**Figure 7:**
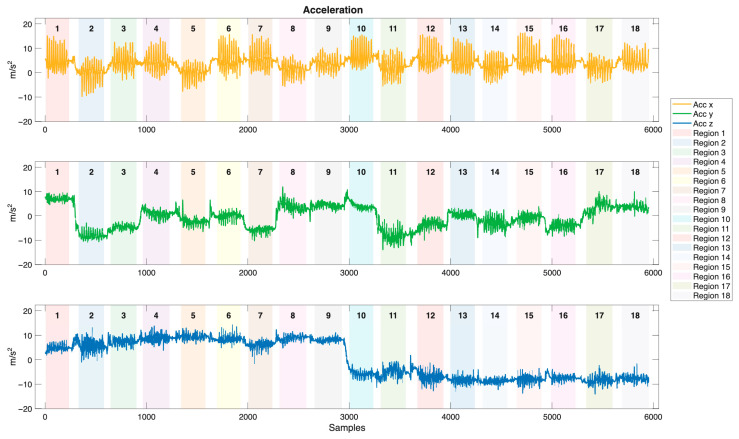
The acceleration signals of the sensor embedded in the toothbrush during a brushing session (m/s^2^).

**Figure 8:**
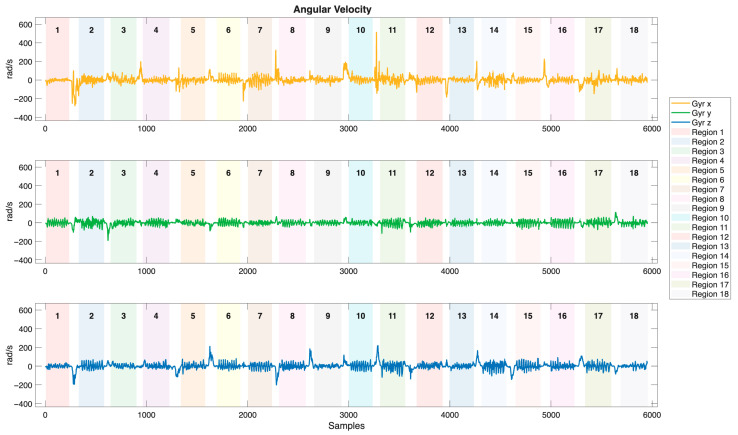
The angular velocity signals of the sensor embedded in the toothbrush during a brushing session (rad/s).

**Figure 9:**
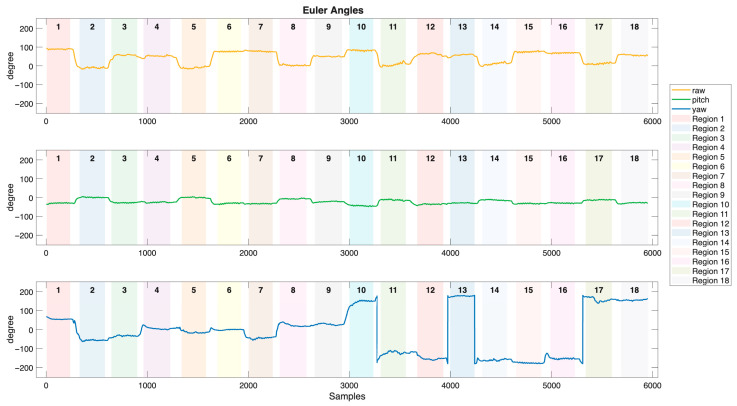
The Euler angle signals of the sensor embedded in the toothbrush during a brushing session (degree).

Tri-axial accelerator, gyroscope, and magnetometer are utilized to capture brushing motion data in this study. The motion signals of acceleration, angular velocity, and Euler angles, collected from the sensor embedded in the toothbrush holder during a brushing session are illustrated in Figure 7, Figure 8 and Figure 9, respectively. Brushing regions are labeled in the figures, with transition segments occurring between each region. The acceleration signals exhibit distinct and periodic vibration patterns across different brushing regions, reflecting both hand movements and vibrations resulting from the interaction between the bristles and the tooth surfaces. The frequency and amplitude of the acceleration serve as critical features for differentiating brushing regions. In contrast, the angular velocity signals are relatively smoother and primarily display obvious peaks during transitions between brushing regions, corresponding to directional changes of the toothbrush. Therefore, angular velocity can effectively capture posture transitions and rotational behaviors throughout the brushing process. Compared to the acceleration and angular velocity signals, the Euler angle signals are more stable and continuous. Within each brushing region, Euler angles exhibit relatively smooth and consistent variations. In contrast, distinct angular fluctuations are observed during transitions between regions, reflecting changes in brushing direction or wrist orientation. By capturing the spatial orientation of the toothbrush, Euler angles provide critical information for distinguishing brushing motions that share similar dynamic characteristics but differ in orientation.

### 3.2. Results of Brushing Region Recognition

In this study, six machine learning classifiers, support vector machine (SVM), Naïve Bayes (NB), K-nearest neighbors (*k*-NN), decision tree (DT), random forest (RF), and adaptive boosting (AdaBoost), are employed to recognize brushing regions based on continuous signals collected from the toothbrush (TB) and wrist sensors (WR). To further investigate the impact of Euler angle features on brushing region recognition, two distinct input data configurations are considered: without and with Euler angles. In addition, a comparison is conducted across three sensor modalities, including TB, WR, and the combination of the toothbrush and wrist sensors (TB + WR).

The average performance of brushing region recognition using various machine learning algorithms and sensor modalities, excluding post-processing approaches in brushing region recognition stage, are presented in Table 4 and Table 5. The reported average results are computed across both configurations (without and with Euler angles). Among all model, RF achieves the highest performance, with an accuracy of 86.23 ± 3.66%, a sensitivity of 83.71 ± 4.52%, a precision of 85.65 ± 4.50%, and an F1-score of 82.96 ± 5.08%, as shown in Table 4. Regarding the average results across different sensor modalities presented in Table 5, TB + WR modality outperforms TB modality and WR modality, achieving an accuracy of 82.91 ± 4.10%, a sensitivity of 80.37 ± 4.54%, a precision of 82.29 ± 4.24%, and an F1-score of 79.72 ± 4.84%.

In comparison with the results presented in Table 4 and Table 5, the average results incorporating post-processing techniques in brushing region recognition stage showed pronounced improvement, as summarized in Table 6 and Table 7. Among all classifiers, *k*-NN demonstrates the most significant enhancement, with accuracy and F1-score increasing by 16.25% and 15.58%, respectively. In terms of sensor modality, WR modality shows the greatest improvement, achieving an accuracy increase of 8.74% and an F1-score improvement of 6.49%. These results indicate that post-processing techniques effectively reduce misclassifications and enhance the overall reliability of brushing region recognition. The detailed brushing region recognition results for all classifiers and sensor modalities with post-processing techniques under two configurations are presented in Table 8 and Table 9.

### 3.2.1. The Performance of Brushing Region Recognition

Table 8 shows the classification performance utilizing only acceleration and angular velocity features. Among all models, the AdaBoost classifier with TB + WR demonstrates the best overall performance, yielding the highest accuracy of 95.39% and F1-score of 94.36%, followed by RF, which reaches an accuracy of 94.76% and an F1-score of 94.79%. DT ranks third, with an accuracy of 92.81% and an F1-score of 94.09%. By comparison, SVM exhibits the lowest performance, reaching accuracies of 66.34%, 62.75%, and 34.46% with TB + WR, TB, and WR modalities, respectively.

In terms of sensor modality, WR yields the lowest performance across all classifiers. Specifically, *k*-NN exhibits an accuracy of 52.74% and an F1-score of 33.76%, while SVM achieves an accuracy of only 34.46% and F1-score of 16.06%. The wrist-mounted sensor captures motion patterns of the arm and wrist during brushing, including forearm trajectories and wrist rotations. These features are effective for identifying broad directional movements, such as distinguishing between the maxilla and mandible or between the left and right quadrants, thereby inferring relative spatial positions of brushing activity. Nevertheless, the WR sensor lacks direct contact with the brush head and the oral cavity, which results in limited sensitivity to subtle variations in brushing angles and bristle-surface interactions. Furthermore, the participant often rotates the toothbrush using finger movements rather than the wrist, particularly when brushing the occlusal and lingual surfaces. As a result, similar kinematic patterns may emerge across different brushing regions, which degrades the discriminative capability of models and reduces classification performance when relying solely on the WR sensor.

On the other hand, using only TB modality demonstrates high classification performance. AdaBoost reaches an accuracy of 94.44% and 93.30% of F1-score. The superior performance is primarily attributed to the motion signals captured by the IMU embedded in the toothbrush, which enable precise characterization of brushing kinematics. Specifically, the TB sensor records both translational and rotational signals derived from raw acceleration and angular velocity, capturing not only the amplitude and frequency of brushing motion but also subtle variations in motion patterns that reflect orientation-related characteristics. These comprehensive motion features provide discriminative information that is essential for accurately recognizing various brushing behaviors. However, despite the anatomical differences between dental surfaces, the combined effects of the wrist compensation mechanisms and the spatial constraints of the oral cavity often result in similar trajectory patterns. Herein, wrist compensation mechanisms refer to natural adjustments in the wrist and elbow to maintain consistent brush-to-tooth contact rather than rotating the toothbrush; likewise, the limited space within the oral cavity restricts the range of achievable motion orientations. Consequently, mirror-symmetric hand movements and comparable toothbrush orientations are frequently observed when brushing anatomically distinct surfaces, such as the mandibular left posterior buccal and the mandibular right posterior lingual surfaces, or the maxillary right posterior buccal and the maxillary left posterior lingual surfaces.

In contrast, TB + WR outperforms the use of either TB or WR alone across all classifiers. DT with TB + WR yields an accuracy of 92.81%, which is slightly higher than that obtained using only TB (91.44%) or WR (74.61%). Similar improvements are observed for *k*-NN, NB, and SVM across all evaluation metrics. The performance improvement stems from the complementary characteristics of the two sensing modalities. Specifically, the TB sensor captures detailed motion dynamics and contact-related variations that are strongly associated with specific brushing surfaces, whereas the WR sensor provides information on forearm and wrist trajectories, which helps disambiguate similar patterns that arise when using TB alone. For example, anatomical constraints may cause two brushing regions to produce nearly identical motion patterns in TB signals; however, the forearm posture and wrist trajectory captured by WR can reveal distinguishing characteristics. By integrating both sensors, the fused modality yields a more discriminative feature set. Furthermore, TB + WR enhances robustness to intra-session variability and reduces misclassifications resulting from mirror-symmetric brushing motions, making it the most reliable approach for fine-grained brushing region recognition.

### 3.2.2. The Performance of Euler Angles-Based Brushing Region Recognition

Table 9 presents the recognition results using different sensor modalities with inputs that include Euler angle features. Among all classifiers, the RF classifier with the combination of the toothbrush and wrist sensors (TB + WR) demonstrates the best overall performance, yielding the highest accuracy of 96.13 ± 2.12%. AdaBoost exhibits the second-best performance with TB + WR modality, achieving an accuracy of 95.58%, a sensitivity of 95.41%, a precision of 94.42%, and an F1-score of 94.67%. By comparison, SVM shows the lowest performance, reaching accuracies of 81.13%, 75.89%, and 48.23% with TB + WR, TB, and WR modalities, respectively.

In comparison with the recognition results presented in Table 8, all classifiers and modalities exhibit enhanced performance with the inclusion of Euler angles. For TB modality, the most substantial increases in accuracy are observed in *k*-NN and SVM, with improvements of 20.93% and 13.14%, respectively. This finding underscores the importance of Euler angles in capturing the spatial orientation of the toothbrush, which is critical for distinguishing distinct brushing surfaces within the oral cavity. Further analysis of confusion matrices reveals that excluding Euler angles leads to increased misclassifications between symmetric brushing regions, such as the maxillary left and right occlusal surfaces, as well as the mandibular left and right occlusal surfaces. These misclassifications can be attributed to the limitations of accelerometer and gyroscope data, which primarily capture translational and rotational movements but lack the capacity to resolve symmetric movements occurring on opposite sides of the oral cavity. In contrast, Euler angles provide essential orientation information that delineates the three-dimensional position of the toothbrush, thus enabling more accurate classification of brushing regions.

On the other hand, SVM exhibits the most pronounced improvement in WR modality, with a 13.77% increase in accuracy. The result underscores the pivotal role of orientation-based features in brushing recognition. Compared to the TB sensor, signals from the WR sensor are more susceptible to noise and ambiguous patterns, as wrist movements often involve compensatory and non-brushing gestures, such as elbow flexion and forearm adjustments. Acceleration and angular velocity features alone are insufficient to resolve spatial ambiguities, particularly when similar motion patterns occur across different brushing regions due to biomechanical overlap, such as between the right maxillary and mandibular buccal surfaces, or between the right maxillary and mandibular lingual surfaces. Furthermore, while angular velocity reflects rotational motion, it lacks the absolute orientation reference. This limitation is particularly critical in wrist-based sensing, where wrist rotation does not necessarily correspond to the actual orientation of the toothbrush relative to the dentition. The consistent improvement in performance across all models in WR modality indicates that Euler angles are not merely supplementary features but essential spatial cues for precise region recognition.

Among all modalities, TB + WR demonstrates the most stable and robust performance. Moreover, SVM exhibits the most pronounced performance improvement, with accuracy and F1-score increasing by 14.79% and 22.52%, respectively. This improvement is primarily due to the enhanced ability to distinguish between spatially similar but anatomically distinct regions when Euler angles are included. Specifically, TB + WR benefits from the complementary motion patterns captured by both sensors, where the TB sensor records fine-grained toothbrush movements, and the WR sensor captures broader forearm trajectories. The inclusion of Euler angles provides crucial orientation context, facilitating the effective fusion of these two sensing modalities.

Moreover, the degree of performance improvement varies substantially among classifiers. Tree-based models, such as DT, RF, and AdaBoost, exhibit smaller gains from the addition of Euler angles compared to non-tree models, including SVM, NB, and *k*-NN. Specifically, the average accuracy improvements across the three modalities for DT, RF, and AdaBoost are 0.64%, 1.36%, and 0.81%, respectively. This stability primarily stems from their inherent ability to leverage hierarchical data partitioning, enabling them to capture complex feature interactions even without strong reliance on explicit spatial orientation features. Additionally, ensemble learning models, such as RF and AdaBoost, maintain robust generalization performance by aggregating multiple learners, thereby reducing variance and mitigating overfitting. In contrast, non-tree models show markedly greater benefits from the inclusion of Euler angles, particularly *k*-NN and SVM, which exhibit average accuracy improvements of 11.33% and 13.90%, respectively. For *k*-NN, which relies on distance metrics in a multidimensional feature space, the inclusion of explicit orientation features prevents brushing patterns from symmetric regions from overlapping in the feature space, thereby reducing misclassification of neighbors. SVM demonstrates the greatest performance improvement among all models due to its reliance on constructing a maximum-margin separating hyperplane. The inclusion of Euler angles effectively enlarges the margin between classes, especially in cases of high intra-class similarity. NB also benefits from the additional orientation cues, achieving an average accuracy improvement of 2.51%. This enhancement is partly attributed to its probabilistic assumptions, which allow it to refine conditional probability estimates when enriched with critical spatial features.

### 3.2.3. The Average Confusion Matrix of Brushing Region Recognition

**Figure 10:**
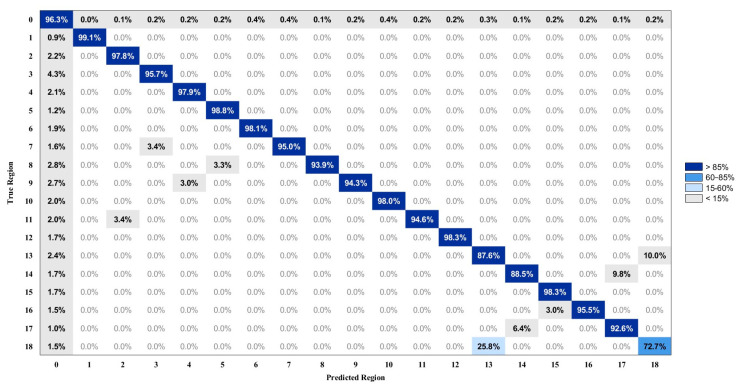
The average confusion matrix of brushing region recognition using the RF model with TB + WR modality.

**Figure 11:**
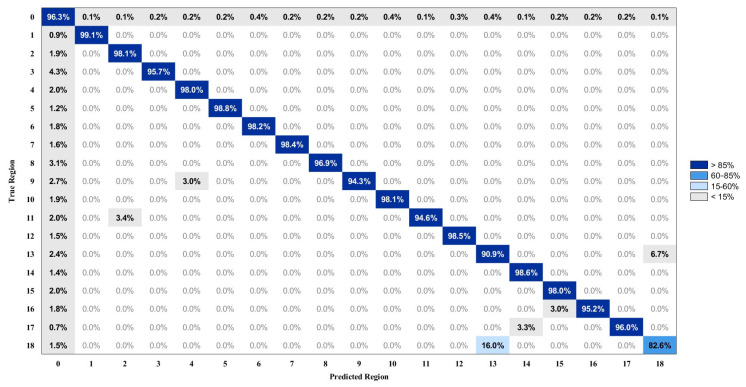
The average confusion matrix of Euler angles–based brushing region recognition using the RF model with TB + WR modality.

Figure 10 and Figure 11 present the average brushing region recognition accuracy using the RF model across 30 sessions. The input features are derived from the combined IMU signals of the toothbrush and the wrist under two feature configurations: without and with Euler angles. Each confusion matrix summarizes the classification performance of 19 defined regions, comprising 18 brushing regions corresponding to the labels illustrated in Figure 4, and an additional transition segment referred to as Region 0. The diagonal elements represent the percentage of correct classifications, while the off-diagonal blocks indicate the percentage of misclassifications.

Figure 10 presents the average confusion matrix derived from input data that only include acceleration- and angular velocity-related features. Overall, the model demonstrates high recognition performance, with classification accuracies exceeding 90% in most regions. Specifically, the classification accuracies for Regions 1, 5, 6, 10, and 15 are 99.1%, 98.8%, 98.1%, 98.0%, and 98.3%, respectively. Despite the overall strong performance, certain regions show misclassification. The confusion rate between Region 14 and Region 17 is 9.8%, between Region 5 and Region 8 is 3.3%, and between Region 3 and Region 7 is 3.4%.

Region 5 and Region 8 are located on the maxillary anterior incisal and lingual surfaces, whereas Region 14 and Region 17 correspond to the mandibular anterior incisal and lingual surfaces, respectively. These regions are situated in the anterior portion of the dental arch, where brushing movements are generally restricted and primarily controlled by subtle wrist rotations rather than large translational displacements. In the absence of an absolute orientation reference, the model depends exclusively on dynamic features captured by the tri-axial accelerometer and gyroscope, particularly angular velocity variations, to differentiate brushing patterns. This limitation poses challenges in distinguishing similar motion trajectories, especially in confined spaces. For instance, greater variability in wrist movement between Regions 14 and 17 produces overlapping angular velocity profiles, resulting in a 9.8% misclassification rate. Likewise, Region 3 corresponds to the maxillary left buccal surface, and Region 7 corresponds to the maxillary right lingual surface. These two regions exhibit comparable wrist rotations and translational movements, leading to overlapping motion patterns.

### 3.2.4. The Average Confusion Matrix of Euler Angles-Based Brushing Region Recognition

In comparison with the average confusion matrix presented in Figure 10, the inclusion of Euler angle features enhances classification accuracy in several regions, as illustrated in Figure 4. For instance, classification accuracy increases by 9.9% in Region 18, 10.1% in Region 14, 3.4% in Region 7, 3.4% in Region 17, and 3.3% in Region 13. Additionally, a decrease in misclassification rates is also observed, with certain confusion rates dropping to 0%. In particular, the confusion rate between Region 14 and Region 17 decreases from 9.8% to 0%, between Region 5 and Region 8 from 3.3% to 0%, and between Region 3 and Region 7 decreases from 3.4% to 0%.

The model demonstrates reliable recognition accuracy with the incorporation of Euler angles, with most regions achieving accuracies above 95%. Specifically, the classification accuracies for Regions 1, 5, 12, and 14 are 99.1%, 98.8%, 98.5%, and 98.6%, respectively, indicating robust performance across these regions. In contrast, Region 18 shows the lowest accuracy at 82.6%, followed by Region 13 with 90.9%. A bidirectional misclassification is observed between these two regions, with 6.7% of Region 13 cases misclassified as Region 18 and 16.0% of Region 18 cases misclassified as Region 13. This confusion is primarily attributed to their anatomical proximity and kinematic similarity in brushing behavior. Both regions are located on the left mandible but different surfaces—Region 13 on the occlusal surface and Region 18 on the lingual surface. The resemblance in wrist rotations and brushing angles likely contributes to classification ambiguity. Analysis of motion data reveals that the roll trajectories of both regions share comparable baseline levels and fluctuation patterns, reflecting similar wrist rotation postures during brushing. The pitch signals overlap in amplitude range and temporal evolution, indicating a consistent inclination of the toothbrush relative to the mandible across the two surfaces. The yaw patterns show relatively minimal variations in both regions, further reducing discriminative power from horizontal rotational movements. Furthermore, the force profiles of Region 13 and Region 18 exhibit overlap, further limiting the separability in feature space. The combination of anatomical adjacency, overlapping motion characteristics, and similar force profiles increases the likelihood of bidirectional misclassification between these regions.

Additionally, partial misclassification of each region into Region 0 is observed in Figure 10 and Figure 11. This phenomenon is likely due to the inherent ambiguity in the spatial or temporal boundaries between adjacent regions, characterized by the absence of well-defined starting points and the existence of gradual inter-region transitions. Such conditions impede the ability of the classifier to establish precise decision boundaries, thereby increasing the likelihood of feature overlap and reducing the overall accuracy of regional discrimination.

## 4. Conclusions

Oral health is a critical component of overall well-being, and proper toothbrushing techniques are crucial for preventing both oral and systemic diseases. To promote and maintain effective oral hygiene, it is essential to develop reliable methods for monitoring brushing behavior. This study proposes a hierarchical approach for recognizing fine-grained brushing regions using two inertial measurement units (IMUs) combined with machine learning algorithms. Various sensor configurations, including TB + WR, TB, and WR, are examined to investigate the contribution of each IMU modality. The TB + WR modality achieves the highest recognition accuracy of 96.13%, outperforming the TB modality (95.25%) and WR modality (81.91%). Additionally, the effectiveness of incorporating Euler angle features into the recognition model is evaluated to assess their impact on classification performance. The results demonstrate the potential and feasibility of the proposed toothbrushing region recognition approach. In future work, the IMU-based recognition approach will be extended by integrating deep learning models such as long short-term memory (LSTM) network and convolutional neural network (CNN). Moreover, brushing duration, surface coverage, and the number of strokes per tooth will be estimated to construct a comprehensive and intelligent toothbrushing monitoring system. To enable system personalization, advanced learning strategies such as transfer learning and fine-tuning will also be explored.

## Figures and Tables

**Table 1:** Region subdivision of the dentition.

Maxilla	Right Posterior Tooth	1. Buccal
		6. Occlusal
		7. Lingual
	Anterior Teeth	2. Labial
		5. Incisal
		8. Lingual
	Left Posterior Tooth	3. Buccal
		4. Occlusal
		9. Lingual
Mandible	Right Posterior Tooth	10. Buccal
		15. Occlusal
		16. Lingual
	Anterior Teeth	11. Labial
		14. Incisal
		17. Lingual
	Left Posterior Tooth	12. Buccal
		13. Occlusal
		18. Lingual

**Table 2:** Feature vectors, where o represents either acceleration (a) or angular velocity (ω).

Feature Vector	Description
$f_{1}\sim f_{14}$	Mean of $o_{x}$, $o_{y}$, $o_{z}$,$\;o_{norm}$, $o_{hori}$, $o_{coro}$, $o_{sagi}$
$f_{15}\sim f_{28}$	Maximum of $o_{x}$, $o_{y}$, $o_{z}$,$\;o_{norm}$, $o_{hori}$, $o_{coro}$, $o_{sagi}$
$f_{29}\sim f_{42}$	Minimum of $o_{x}$, $o_{y}$, $o_{z}$,$\;o_{norm}$, $o_{hori}$, $o_{coro}$, $o_{sagi}$
$f_{43}\sim f_{56}$	Variance of $o_{x}$, $o_{y}$, $o_{z}$,$\;o_{norm}$, $o_{hori}$, $o_{coro}$, $o_{sagi}$
$f_{57}\sim f_{70}$	Standard deviation of $o_{x}$, $o_{y}$, $o_{z}$,$\;o_{norm}$, $o_{hori}$, $o_{coro}$, $o_{sagi}$
$f_{71}\sim f_{84}$	Kurtosis of $o_{x}$, $o_{y}$, $o_{z}$,$\;o_{norm}$, $o_{hori}$, $o_{coro}$, $o_{sagi}$
$f_{85}\sim f_{98}$	Skewness of $o_{x}$, $o_{y}$, $o_{z}$,$\;o_{norm}$, $o_{hori}$, $o_{coro}$, $o_{sagi}$
$f_{99}\sim f_{112}$	Range of $o_{x}$, $o_{y}$, $o_{z}$,$\;o_{norm}$, $o_{hori}$, $o_{coro}$, $o_{sagi}$

**Table 3:** Euler angles-based feature vectors, where o represents either acceleration (a) or angular velocity (ω).

Feature Vector	Description
$f_{1}\sim f_{17}$	Mean of $o_{x}$, $o_{y}$, $o_{z}$,$\;o_{norm}$, $o_{hori}$, $o_{coro}$, $o_{sagi}$, Pitch, Roll, Yaw
$f_{18}\sim f_{34}$	Maximum of $o_{x}$, $o_{y}$, $o_{z}$,$\;o_{norm}$, $o_{hori}$, $o_{coro}$, $o_{sagi}$, Pitch, Roll, Yaw
$f_{35}\sim f_{51}$	Minimum of $o_{x},o_{y},o_{z},o_{norm}$, $o_{hori}$, $o_{coro}$, $o_{sagi}$, Pitch, Roll, Yaw
$f_{52}\sim f_{68}$	Variance of $o_{x}$, $o_{y}$, $o_{z}$,$\;o_{norm}$, $o_{hori}$, $o_{coro}$, $o_{sagi}$, Pitch, Roll, Yaw
$f_{69}\sim f_{85}$	Standard deviation of $o_{x}$, $o_{y}$, $o_{z}$, $\omega_{z}$,$\;o_{norm}$, $o_{hori}$, $o_{coro}$, $o_{sagi}$, Pitch, Roll, Yaw
$f_{86}\sim f_{102}$	Kurtosis of $o_{x}$, $o_{y}$, $o_{z}$,$\;o_{norm}$, $o_{hori}$, $o_{coro}$, $o_{sagi}$, Pitch, Roll, Yaw
$f_{103}\sim f_{119}$	Skewness of $o_{x}$, $o_{y}$, $o_{z}$,$\;o_{norm}$, $o_{hori}$, $o_{coro}$, $o_{sagi}$, Pitch, Roll, Yaw
$f_{120}\sim f_{136}$	Range of $o_{x}$, $o_{y}$, $o_{z}$,$\;o_{norm}$, $o_{hori}$, $o_{coro}$, $o_{sagi}$, Pitch, Roll, Yaw

**Table 4:** The average performance using different machine learning models without post-processing techniques (unit: %).

Classifier	Accuracy	Sensitivity	Precision	F1-Score
SVM	60.61 ± 6.54	55.08 ± 5.26	57.22 ± 6.09	52.05 ± 5.45
NB	77.12 ± 4.21	71.92 ± 5.25	75.70 ± 5.13	71.04 ± 5.68
*k*-NN	54.32 ± 4.53	44.38 ± 4.90	44.76 ± 4.71	43.15 ± 4.73
DT	78.66 ± 4.08	74.88 ± 4.91	75.78 ± 4.80	74.11 ± 5.45
RF	**86.23 ± 3.66**	**83.71 ± 4.52**	**85.65 ± 4.50**	**82.96 ± 5.08**
AdaBoost	85.58 ± 3.75	82.83 ± 4.60	84.57 ± 4.56	82.20 ± 5.11

Note: bold fonts represent the best performance.

**Table 5:** The average performance using different sensor modalities without post-processing techniques (unit: %).

Modality	Accuracy	Sensitivity	Precision	F1-Score
TB + WR	**82.91 ± 4.10**	**80.37 ± 4.54**	**82.29 ± 4.24**	**79.72 ± 4.84**
TB	80.63 ± 3.64	77.88 ± 4.20	79.58 ± 3.99	77.08 ± 4.39
WR	57.71 ± 5.64	48.15 ± 5.8	49.98 ± 6.67	45.95 ± 6.51

Note: bold fonts represent the best performance.

**Table 6:** The average performance using different machine learning models with post-processing techniques (unit: %).

Classifier	Accuracy	Sensitivity	Precision	F1-Score
SVM	61.80 ± 15.58	56.35 ± 14.49	48.66 ± 16.09	49.76 ± 16.07
NB	82.93 ± 6.65	79.27 ± 8.44	74.77 ± 10.65	75.87 ± 9.90
*k*-NN	70.57 ± 8.08	65.02 ± 9.95	56.09 ± 11.01	58.73 ± 10.72
DT	86.61 ± 6.46	85.03 ± 7.98	80.31 ± 10.77	81.76 ± 9.85
RF	**90.42 ± 4.44**	**89.04 ± 5.54**	**85.70 ± 7.68**	**86.66 ± 6.93**
AdaBoost	89.97 ± 4.26	88.47 ± 5.36	84.92 ± 7.36	85.98 ± 6.64

Note: bold fonts represent the best performance.

**Table 7:** The average performance using different sensor modalities with post-processing techniques (unit: %).

Modality	Accuracy	Sensitivity	Precision	F1-Score
TB + WR	**88.57 ± 6.43**	**87.20 ± 7.23**	**84.39 ± 9.59**	**84.98 ± 8.82**
TB	85.76 ± 6.90	84.56 ± 7.64	80.90 ±9.60	81.71 ± 9.03
WR	66.64 ± 9.41	59.83 ± 11.01	49.93 ± 12.58	52.69 ± 12.21

Note: bold fonts represent the best performance.

**Table 8:** The performance of brushing region recognition (unit: %).

Classifier	Modality	Accuracy	Sensitivity	Precision	F1-Score
SVM	TB + WR	66.34 ± 14.71	61.56 ± 13.81	52.68 ± 17.16	54.42 ± 16.60
	TB	62.75 ± 23.08	59.49 ± 21.32	51.69 ± 21.51	52.74 ± 22.50
	WR	34.46 ± 12.37	24.92 ± 9.73	14.59 ± 7.61	16.06 ± 8.76
NB	TB + WR	89.85 ± 5.27	87.86 ± 6.75	86.40 ± 9.42	86.40 ± 8.44
	TB	89.65 ± 4.95	88.16 ± 6.20	85.80 ± 8.57	86.28 ± 7.70
	WR	65.51 ± 10.59	57.00 ± 13.56	45.67 ± 15.96	48.98 ± 15.20
*k*-NN	TB + WR	78.97 ± 8.92	75.10 ± 11.21	67.39 ± 13.22	69.80 ± 12.63
	TB	63.01 ± 8.74	56.46 ± 11.01	45.59 ± 11.28	48.77 ± 11.23
	WR	52.74 ± 10.91	41.87 ±13.25	30.58 ± 12.83	33.76 ± 13.15
DT	TB + WR	92.81 ± 5.14	92.68 ± 6.05	90.45 ± 9.02	91.09 ± 8.03
	TB	91.44 ± 4.72	91.14 ± 5.57	88.24 ± 8.20	89.08 ± 7.30
	WR	74.61 ± 9.85	69.97 ± 12.76	60.91 ± 14.82	63.78 ± 14.27
RF	TB + WR	94.76 ± 2.82	94.47 ± 3.40	93.04 ± 5.64	93.41 ± 4.77
	TB	94.11 ± 2.90	93.90 ± 3.53	92.33 ± 5.27	92.76 ± 4.55
	WR	80.34 ± 7.51	76.27 ± 9.29	67.90 ± 12.37	70.45 ± 11.38
AdaBoost	TB + WR	**95.39 ± 2.96**	**95.09 ± 3.72**	**94.20 ± 5.84**	**94.36 ± 5.03**
	TB	94.44 ± 3.61	94.27 ± 4.51	92.97 ± 6.12	93.30 ± 5.50
	WR	78.87 ± 6.93	74.46 ± 8.87	65.57 ± 11.23	68.38 ±10.46

Note: bold fonts represent the best performance.

**Table 9:** The performance of Euler angles-based brushing region recognition (unit: %).

Classifier	Modality	Accuracy	Sensitivity	Precision	F1-Score
SVM	TB + WR	81.13 ± 18.16	79.42 ± 18.00	77.29 ± 21.16	76.94 ± 20.30
	TB	75.89 ± 14.73	74.75 ± 14.66	70.75 ± 19.09	70.45 ± 17.92
	WR	48.23 ± 10.47	37.94 ± 9.45	24.98 ± 9.99	27.92 ± 10.36
*k*-NN	TB + WR	86.05 ± 4.98	84.16 ± 6.25	78.65 ± 8.70	80.35 ± 7.81
	TB	83.94 ± 5.58	83.22 ± 6.57	77.03 ± 8.92	78.89 ± 8.19
	WR	58.73 ± 9.33	49.33 ± 11.43	37.30 ± 11.17	40.82 ± 11.34
NB	TB + WR	92.79 ± 4.06	91.51 ± 4.06	91.82 ± 6.66	91.20 ± 6.12
	TB	91.45 ± 3.80	90.72 ± 5.04	88.75 ± 6.65	89.14 ± 6.11
	WR	68.31 ± 11.22	60.39 ± 13.96	50.16 ± 16.62	53.18 ± 15.85
DT	TB + WR	93.10 ± 5.38	93.08 ± 6.40	90.84 ± 9.50	91.54 ± 8.41
	TB	92.35 ± 5.16	92.41 ± 6.44	89.87 ± 9.33	90.63 ± 8.37
	WR	75.32 ± 8.50	70.92 ± 10.64	61.57 ± 13.73	64.46 ± 12.72
RF	TB + WR	**96.13 ± 2.12**	**96.10 ±2.70**	**95.51 ± 3.89**	**95.60 ± 3.42**
	TB	95.25 ± 2.74	95.35 ± 3.49	94.31 ± 4.97	94.56 ± 4.42
	WR	81.91 ± 8.54	78.18 ± 10.86	71.08 ± 13.92	73.19 ± 13.05
AdaBoost	TB + WR	95.58 ± 2.62	95.41 ± 3.36	94.42 ± 4.88	94.67 ± 4.32
	TB	94.89 ± 2.73	94.82 ± 3.40	93.51 ± 5.32	93.87 ± 4.59
	WR	80.67 ± 6.73	76.76 ± 8.32	68.84 ± 10.75	71.31 ± 9.94

Note: bold fonts represent the best performance.

## Data Availability

The data presented in this study are available on request from the corresponding author. The data are not publicly available due to privacy and ethical restrictions.
